# Towards an effective sensing technology to monitor micro-scale interface loosening of bioelectronic implants

**DOI:** 10.1038/s41598-021-82589-3

**Published:** 2021-02-10

**Authors:** Marco P. Soares dos Santos, Rodrigo Bernardo, Luís Henriques, A. Ramos, Jorge A. F. Ferreira, Edward P. Furlani, A. Torres Marques, José A. O. Simões

**Affiliations:** 1 Centre for Mechanical Technology & Automation, TEMA, University of Aveiro, 3810-193 Aveiro, Portugal; 2 Department of Mechanical Engineering, University of Aveiro, 3810-193 Aveiro, Portugal; 3 Associated Laboratory for Energy, Transports and Aeronautics (LAETA), 4150-179 Porto, Portugal; 4 Department of Chemical and Biological Engineering, University at Buffalo, SUNY, Buffalo, NY USA; 5 Department of Electrical Engineering, University at Buffalo, SUNY, Buffalo, NY USA; 6 Mechanical Engineering Department, University of Porto, 4200-465 Porto, Portugal

**Keywords:** Biomedical engineering, Computational biophysics, Mechanical engineering

## Abstract

Instrumented implants are being developed with a radically innovative design to significantly reduce revision surgeries. Although bone replacements are among the most prevalent surgeries performed worldwide, implant failure rate usually surpasses 10%. High sophisticated multifunctional bioelectronic implants are being researched to incorporate cosurface capacitive architectures with ability to deliver personalized electric stimuli to peri-implant target tissues. However, the ability of these architectures to detect bone-implant interface states has never been explored. Moreover, although more than forty technologies were already proposed to detect implant loosening, none is able to ensure effective monitoring of the bone-implant debonding, mainly during the early stages of loosening. This work shows, for the first time, that cosurface capacitive sensors are a promising technology to provide an effective monitoring of bone-implant interfaces during the daily living of patients. Indeed, in vitro experimental tests and simulation with computational models highlight that both striped and circular capacitive architectures are able to detect micro-scale and macro-scale interface bonding, debonding or loosening, mainly when bonding is weakening or loosening is occurring. The proposed cosurface technologies hold potential to implement highly effective and personalized sensing systems such that the performance of multifunctional bioelectronic implants can be strongly improved. Findings were reported open a new research line on sensing technologies for bioelectronic implants, which may conduct to great impacts in the coming years.

## Introduction

The widespread of bone replacement surgeries highlights their worldwide societal impact in minimizing the burden of musculoskeletal disorders^1–4^. Although arthroplasties are nowadays among the most prevalent surgeries performed around the world, significant increasing incidences are predicted in the forthcoming decades^1,2,5–7^. Despite relevant advances in implant technology have emerged throughout the last two decades, implant failures usually surpass 10%^8–10^ and their survival rate may decrease to 60% at 25 years after primary arthroplasty^1,11,12^. The inability of the current implant technology to ensure a revision-free performance is even more relevant as around 30% of the overall patients are young and this number is estimated to increase up to 60% by 2030^4,13–15^.

The use of uncemented bone-implant fixations is a worldwide increasing trend, mainly in younger and/or active patients^16,17^. However, uncemented technologies are not effective to avoid bone loss due to stress-shielding. This is a mechanical phenomenon due to the delivery of mechanical stimuli patterns along the peri-implant regions that are different to those delivered along natural bones (no implant scenario)^5,17^. Unstable bone-implant fixations are significantly promoted by stress-shielding effects, which can ultimately result in late implant failure due to aseptic loosening^17,18^. The implant loosening is the most common indication for revision surgeries, currently with incidences that can exceed 50%^1,4,6,8^.

**Figure 1:**
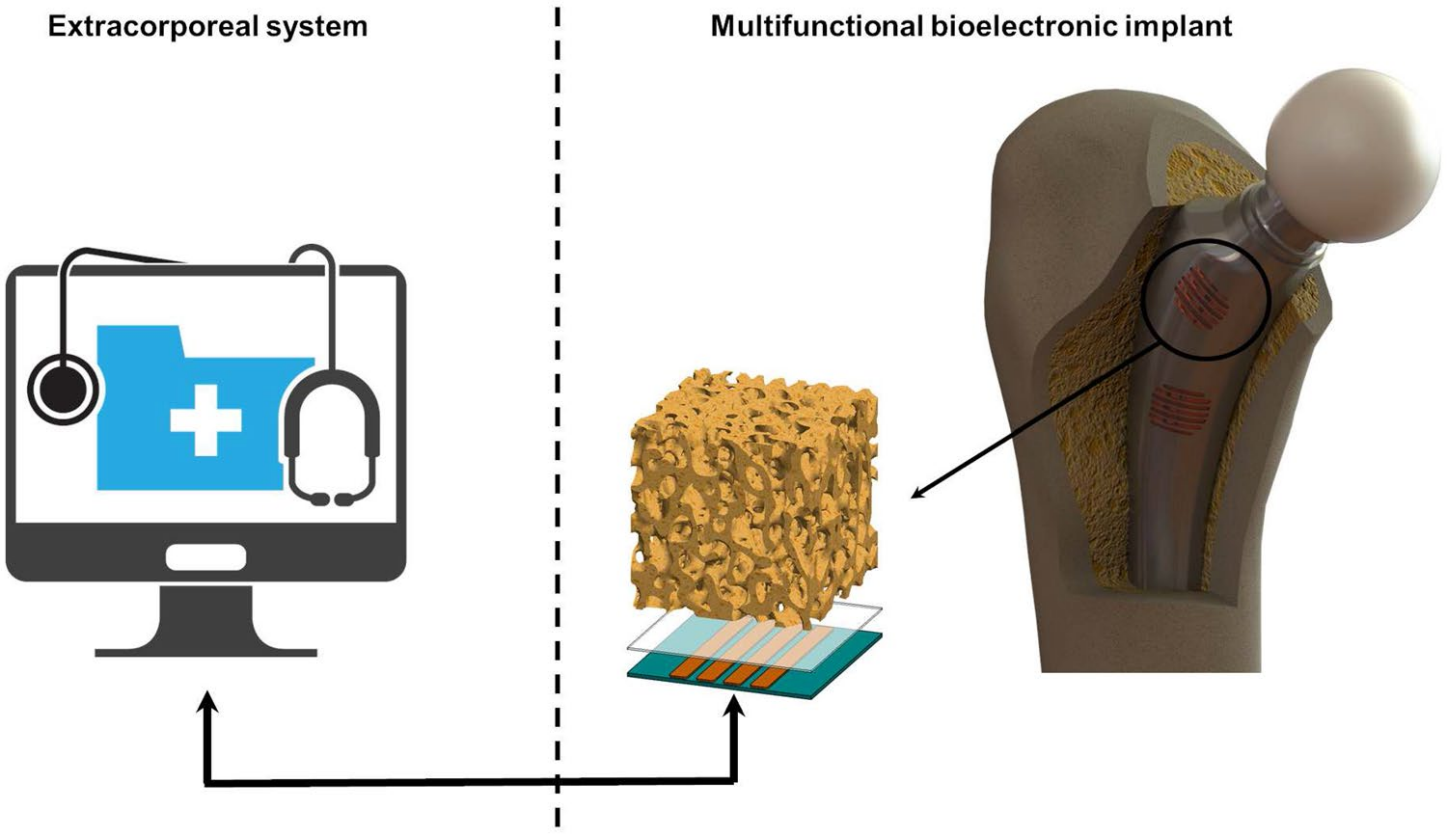
Bioelectronic implants incorporating cosurface capacitive architectures for therapeutic actuation and micro-scale sensing, both extracorporeally controlled by clinicians.

The field of bioelectronic medicine has recently attracted a lot of research and development activity^19–22^. An effective monitoring of the bone-implant loosening is mandatory to develop high sophisticated implantable biodevices with ability to minimize revision procedures^5,19,23–25^. The development of instrumentation to be incorporated inside bone implants was firstly carried out only to measure some biomechanical quantities in vivo, including in human patients, such as forces, moments, deformations and temperatures^6,26–29^. These instrumented implants also incorporate wireless communication and non-autonomous powering systems^27–29^. Nevertheless, they were not designed to be highly personalized bioelectronic medical devices to control the bone-implant responses and integration, since this requirement will be only fulfilled by incorporating therapeutic actuation systems, to control the peri-implant bone growth, and sensing systems, to monitor the bone-implant interface states^5,23^. Significant breakthroughs have been carried out to develop personalized instrumented bioelectronic implants comprising bone-implant loosening sensors and biophysical delivery systems, both with ability to be extracorporeally controlled by clinicians as long as required to avoid implant failures (Fig. 1)^5,19,23^. Recent findings provide strong motivation and justification to further investigate biophysical therapeutic actuators based on the delivery of electromagnetic stimuli^5,19,30–33^. The use of cosurface capacitive stimulators is innovative as: (1) they are able to deliver electric stimuli using electrodes in the same surface (whatever the surface topology); (2) they may comprise as many electrodes as required; (3) their geometry can be customized for each peri-implant region, allowing miniaturized, stretchable and flexible integration inside bioelectronic implants; (4) each electrode can be independently controlled (excitation magnitude, frequency, daily stimulation time, overall stimulation duration, etc.) according to stimulation therapies defined by clinicians for personalized stimulation of target peri-implant tissues; and (5) they are non-complex and cost-effective systems^5,19,34^. Nevertheless, the ability of these architectures to provide high effective bone-implant loosening sensing has never been explored^35,36^. If the same capacitive architectures could be used both as sensors and actuators, then bioelectronic implants would be more easily designed and the same peri-implant target tissues would be more effectively controlled. Five methodologies have already been proposed to monitor time-dependent loosening of bone-implant interfaces: vibrometric, acoustic, bioelectric impedance, magnetic induction, and strain^35^. Although around forty sensing technologies were already developed (not including imaging technologies), none is suitable for incorporation inside multifunctional bioelectronic implants ensuring effective sensing during the daily living of patients^35^. Indeed, current loosening sensing technologies are rather limited, given that: (i) they are only able to detect few loosening stages^35,37^; (ii) they fail to detect early states of loosening^35,38,39^; (iii) their ability to identify region- and magnitude-dependent loosening is very reduced^25,35^; (iv) they require extracorporeal excitations to drive the sensing system, and thus the clinical follow-up cannot be performed throughout the daily life of patients^35,40,41^. This follow-up must be performed to detect early loosening (micro-scale sizes), intermediate loosening (ideally a large number of intermediate loosening sizes), and late (massive) loosening^38^. Besides, bone resorption must be monitored to identify if it is linear (equally distributed around the implant) or focal (focused on specific regions of the bone-implant interface). We here hypothesized that cosurface capacitive technologies are able to monitor bone-implant fixation states. Our verification method allows to conclude that this hypothesis is valid in vitro. Therefore, breakthroughs achieved in this work include a unique insight into the in vitro performance of cosurface capacitive architectures as sensing systems to detect bone-implant loosening. Successful results were obtained in experimental in vitro tests and numerical simulations. Their new functions in the scope of bioelectronic medicine aim to contribute towards the design of the future multifunctional implantable medical devices.

## Results

### Capacitive architectures

**Figure 2:**
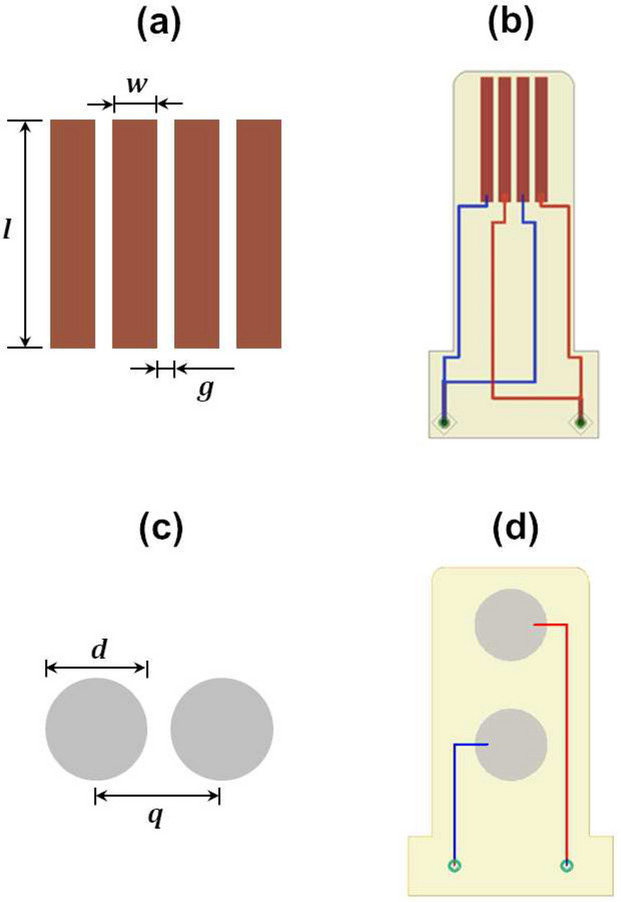
Planar capacitive architectures: (**a**) Design of the striped sensor ($l=10$ mm; $w=1$ mm; $g=0.5$ mm); (**b**) Electrical schematic of the striped sensor; (**c**) Design of the circular sensor ($d=6$ mm; $q=10$ mm); (**d**) Electrical schematic of the circular sensor.

Considering different architectures, geometries and materials, we designed and fabricated two cosurface capacitive sensors:

- Striped architecture: it comprises four electrodes with 10 mm length, 1 mm wide and 0.1 mm thick, 0.5 mm apart from each other (Fig. 2a). The overall sensor dimensions are $10\times 5.5\times 0.1$ mm. It was engineered as two parallel-connected capacitors comprising two negatively charged electrodes and two positively charged electrodes (Fig. 2b). Copper electrodes were used to exhibit very high electrical conductivity and negligible magnetic properties.
- Circular architecture: it comprises two electrodes with 6 mm diameter and 1.5 mm thick, 10 mm equidistant from each other (Fig. 2c). This sensor dimensions are $16\times 6\times 1.5$ mm. One electrode was negatively charged, while the other was positively charged (Fig. 2d). Iron-nickel (Ni-Fe alloy) electrodes were used to exhibit ferromagnetic properties and lower electrical conductivity comparing to copper materials.

### Monitoring platform

A 24-bit capacitance-to-digital converter was used to measure capacitance changes for different bone-sensor interfaces (see “Methods” section). A voltage excitation, defined as a 32 kHz square waveform with 5 V amplitude, was delivered to the electrodes.

### Experimental setup

**Figure 3:**
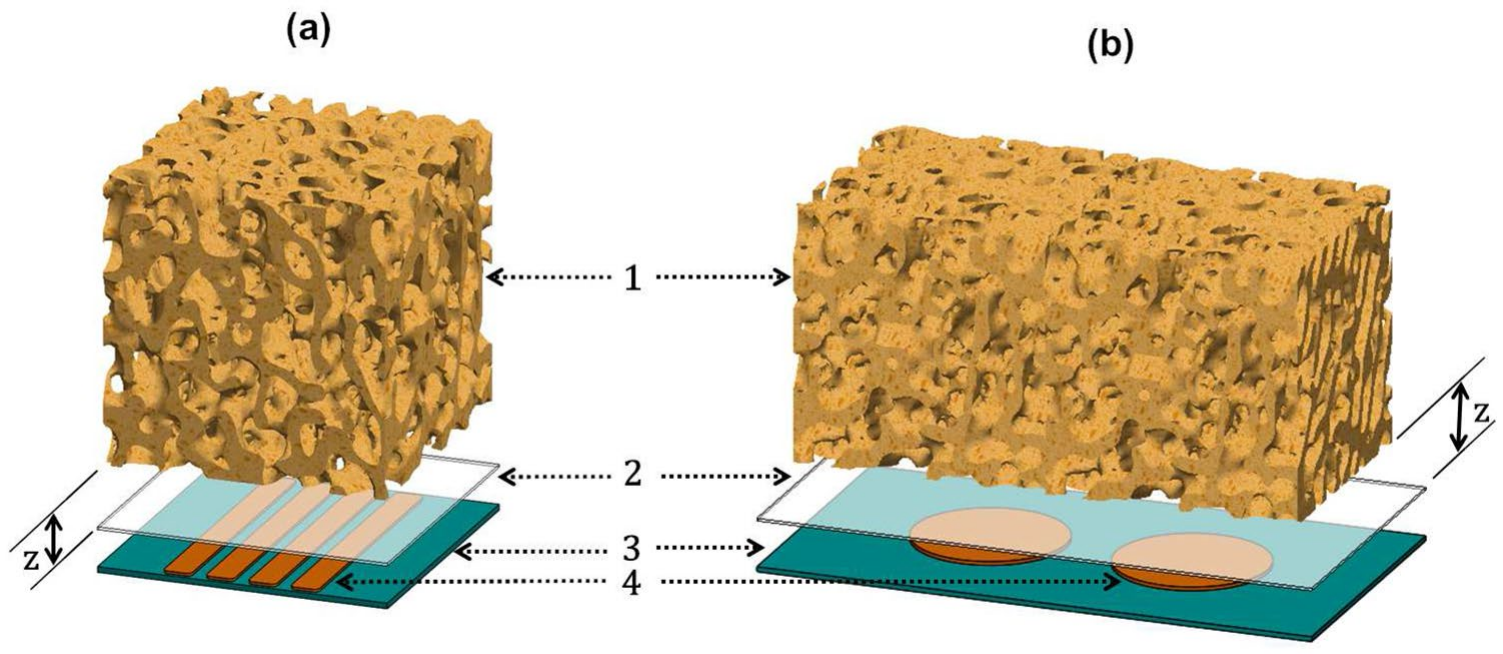
Sensing interfaces: (**a**) using the striped capacitive architecture; (**b**) using the circular capacitive architecture. Legend: 1—trabeculae; 2—interfacial sheet; 3—substrate; 4—electrodes; *z*—vertical displacement between the bone samples and the capacitive architectures.

An experimental apparatus was designed to provide an approximative scenario of peri-prosthetic bone boss, as well as to provide a sensing scenario without increasing the risk of weakening the bone-implant fixation. Two bone-sensor interfaces were implemented by positioning 1 mm thick polymeric sheets between the sensors and the bone samples to prohibit bone-electrode contacts and ensure very high electrical resistivity, as illustrated in Fig. 3a,b. These electrically isolated sheets were positioned above the sensors, and bone samples in different vertical positions above the sheets, as these bone-sensor interfaces were designed to mimic how sensors must be positioned inside multifuncional bioelectronic implants, as close as possible to the implant’s surface. These interfaces are similar to those that have been proposed for therapeutic actuators incorporated inside such implants to deliver electromagnetic stimuli to bone structures^5,19,30^. Notice that this encapsulation thickness, provided by the polymeric sheets, can be customized according to material properties, voltage powering the electrodes, etc.

**Figure 4:**
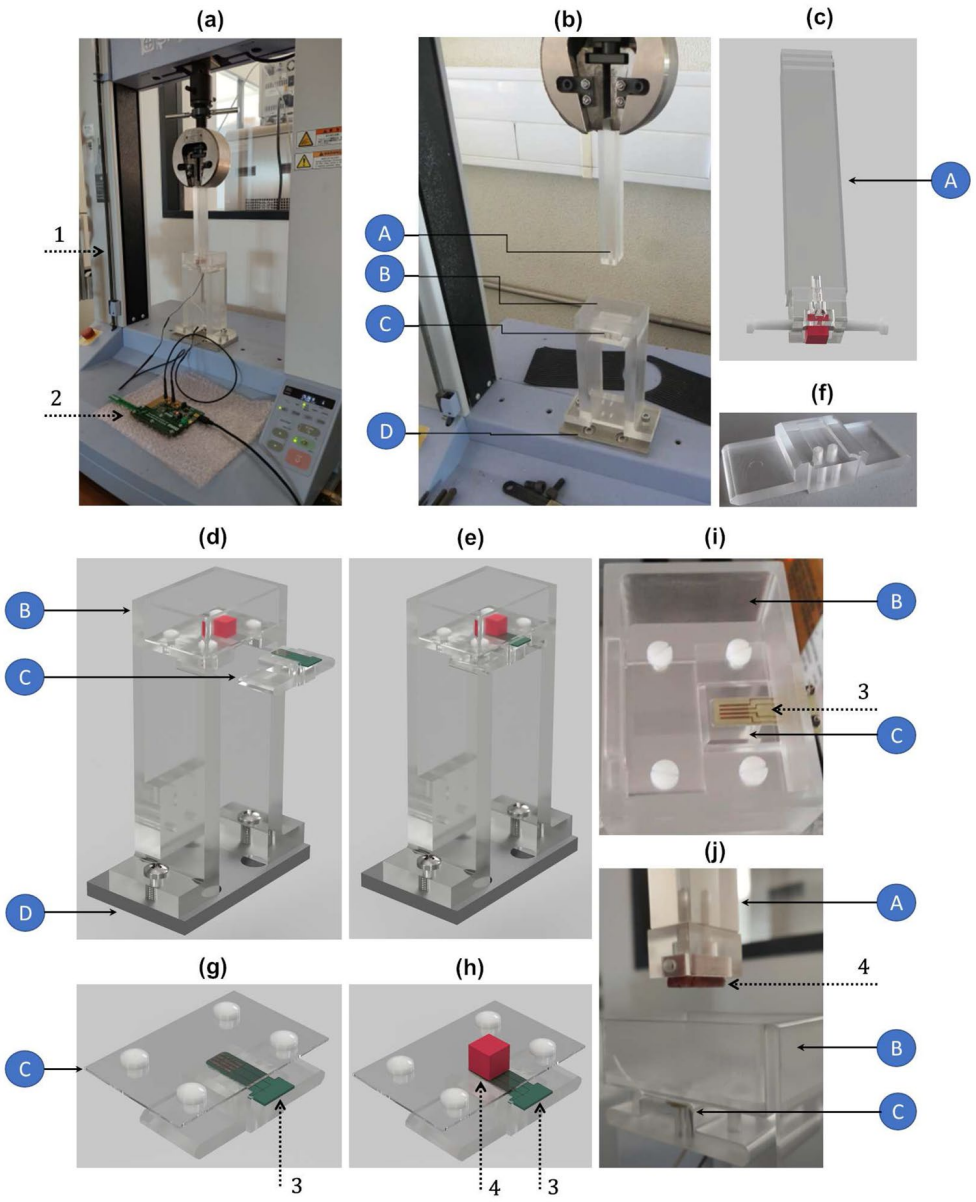
Experimental apparatus implemented for experimental tests in vitro: (**a**) Overall experimental apparatus; (**b**) Additional structures manufactured to implement different bone-sensor interfaces: A—polymeric stem to attach each bone sample; B—polymeric structure to attach each sensor and related interfacial sheet; C—polymeric board where the sensor is positioned (the assembly is incorporated within structure B); D—aluminium board; (**c**) Detailed design of the polymeric structure A; (**d**,**e**) Detailed design of the polymeric structure B, including the polymeric structure C; (**f**) Detailed design of the cavity within the structure C where the PCB board is positioned; (**g**,**h**) Detailed design of the polymeric structure C, including the interfacial sheet; (**i**) Sensor and structure C assembled into the structure B; (**j**) A bone-sensor interface in a non-contact scenario. Legend: 1—testing machine; 2—capacitance-to-digital converter; 3—capacitive architecture (striped or circular); 4—bone sample.

The overall experimental apparatus is shown in Fig. 4a. An universal testing machine was used: (i) to provide vertical motion (step displacements) of the bone sample such that different bone-sensor interfaces can be established; (ii) to perform compression and decompression tests (step forces) for contact scenarios, i.e., when mechanical contacts occur between the bone sample and the interfacial sheet. The structure A (Fig. 4b,c) was clamped to the moving structure (upper part) of the testing machine. A hollowed region, shaped as a $20\times 10\times 10$ mm rectangular cuboid, was designed on the bottom extremity of this stem, where each bone sample was attached using two nylon screws. The structure B (Fig. 4b,d) was machined to provide two main functions: (1) to ensure an accurate and fixed positioning of the capacitive sensor and interfacial sheet in the lower part of the testing machine; (2) to incorporate an artificial vision system to characterize the contact scenarios occurring for different bone-sensor interfaces. Each sensor was accurately positioned within a 2 mm thick cavity of structure C (Fig. 4b,f–h) and two nylon screws were used to clamp each capacitive circuitry to the interfacial sheet. The assembly (sensor and structure C) was then screwed to the structure B (Fig. 4e,i). Figure 4j highlights a bone-sensor interface without bone-sheet contact simulating a bone-implant loosening state. Structures A, B and C were designed using a polymeric material (PMMA) to provide an electrically isolating apparatus and to minimize the impact of electromagnetic fields produced by the testing machine on capacitance changes for different bone-sensor interfaces.

**Figure 5:**
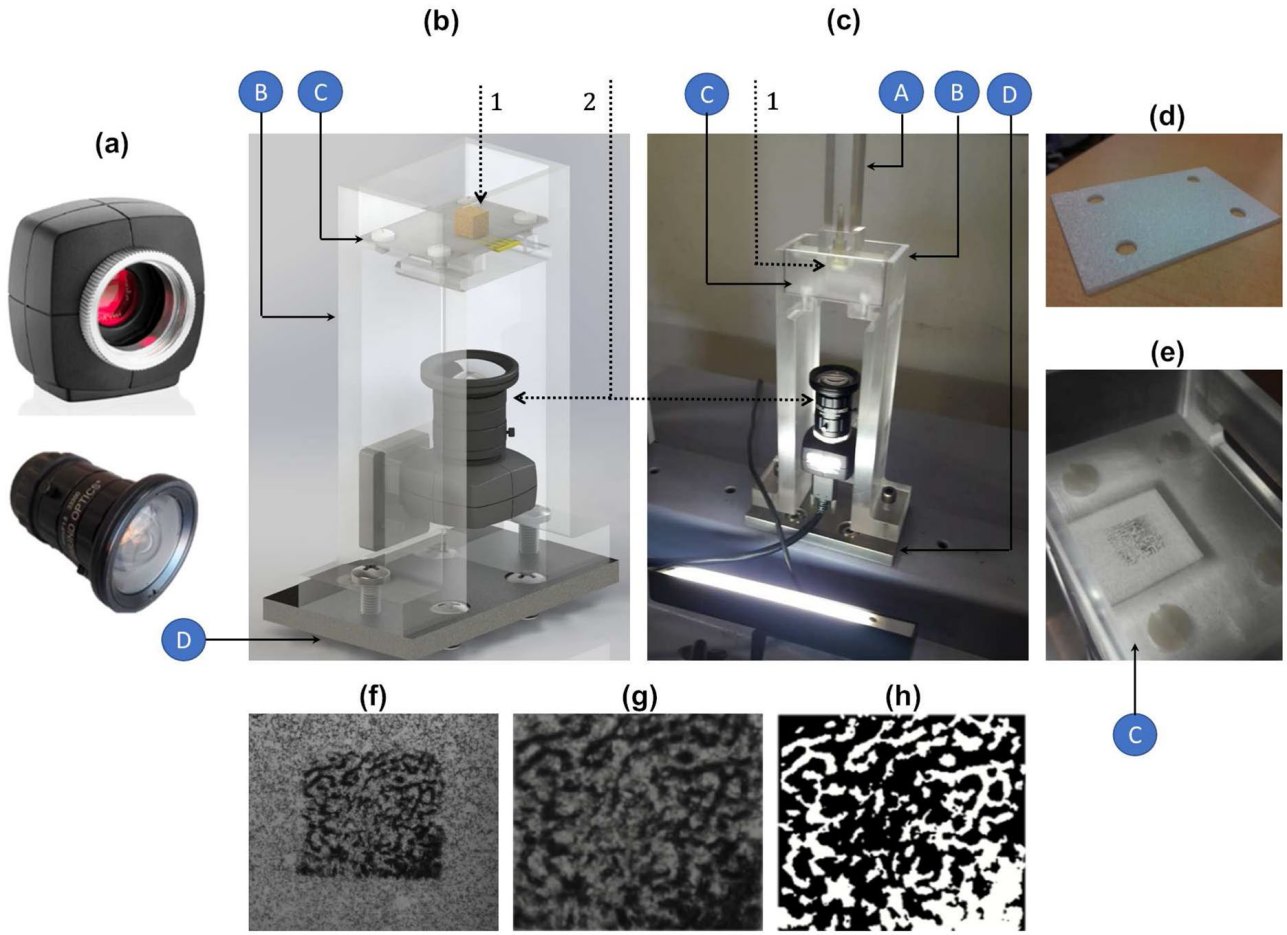
Method to characterize different bone-sensor interfaces in vitro: (**a**) Camera and lens of the artificial vision system; (**b**,**c**) Structure B (Fig. 4d,f) incorporating the artificial vision system in a contact scenario; (**d**) Coating of the polymeric sheet with metallic powder; (**e**) Contact pattern stamped on the polymeric sheet after removing the bone sample; (**f**) Contact pattern acquired by the vision system; (**g**) Image processing before binarization; (**h**) Image processing after binarization. Legend: A—polymeric stem to attach each bone sample; B—polymeric structure to attach each sensor and related interfacial sheet; C—polymeric board where the sensor is positioned; D—aluminium board; 1—bone sample; 2—camera and lens.

The characterization of the different bone-sensor interfaces for contact scenarios was performed using an artificial vision system (Fig. 5a) composed by a high resolution camera and a lens with 4 mm fixed focal length. Both were embedded within the structure B (Fig. 5b,c). The bone-sheet contact patterns were obtained by coating the upper surface of the polymeric sheet with metallic powder, as shown in Fig. 5d. Contact patterns were observed when trabeculae from the bone samples bonded with particles of the metallic powder (Fig. 5e). Different patterns are obtained by performing 10 $\upmu \text {m}$ step vertical motions of decalcified bone samples. All contact patterns for different bone-sensor interfaces were acquired (Fig. 5f). Image processing was performed to reduce noise using a gaussian smoothing filter, as well as to binarize all contact patterns with an adaptive threshold (threshold computed considering the local mean intensity in the neighbourhood of each pixel) (Fig. 5g,h).

### Bone samples

**Figure 6:**
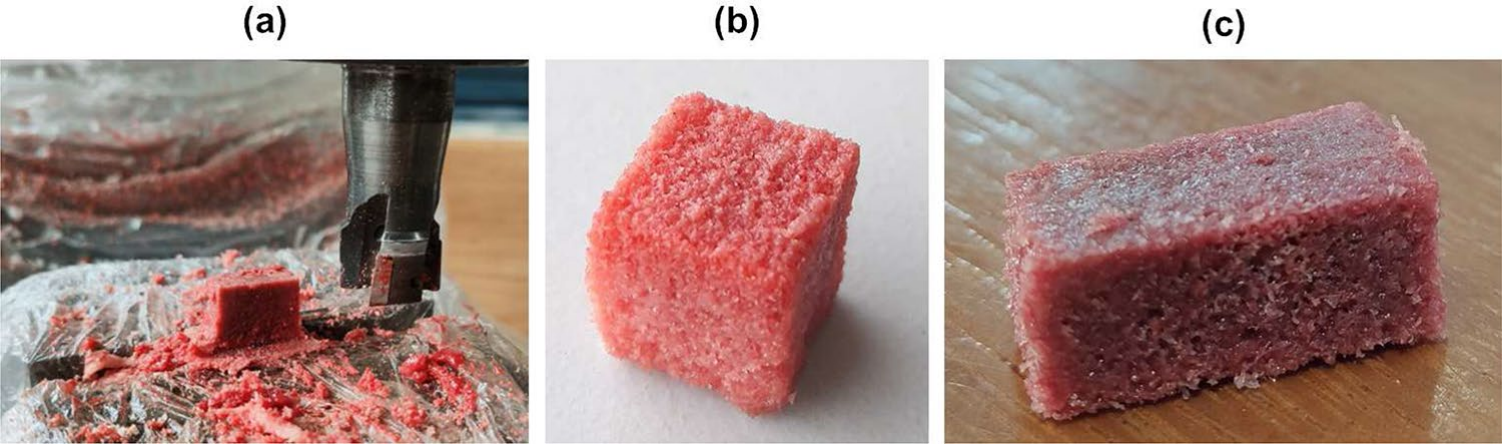
Cadaveric bone samples used in experimental in vitro tests: (**a**) machining of the porcine bone samples; (**b**) bone samples used to analyse the striped architecture; (**c**) bone samples used to analyse the circular architecture.

Twenty-two trabecular samples from cadaveric distal femoral epiphysis of post-mortem porcine models (under 1 year) were machined, as shown in Fig. 6a. Eight cubic-shaped samples with $10\times 10\times 10\,\text {mm}$ and 8 rectangular cuboid-shaped samples with $20\times 10\times 10\,\text {mm}$ were used to experimentally analyse the performance of the striped and circular architectures, respectively (Fig. 6b,c).

Decalcification and preservation of trabecular microstructures of six bone samples (three cubic-shaped samples and three rectangular cuboid-shaped samples) were carried out before characterization of the bone-sheet contact patterns. Non-decalcified bone mainly consists in a biphasic structure composed by $\approx 65\%$ mineral and $\approx 35\%$ organic phases^42^. After bone decalcification, bone becomes a soft tissue mainly comprising a $\approx 35\%$ organic phase and a liquid phase that may fulfil up to $\approx 65\%$ of the decalcified structure (percentage related to the drying time).

### Experimental tests in vitro

**Figure 7:**
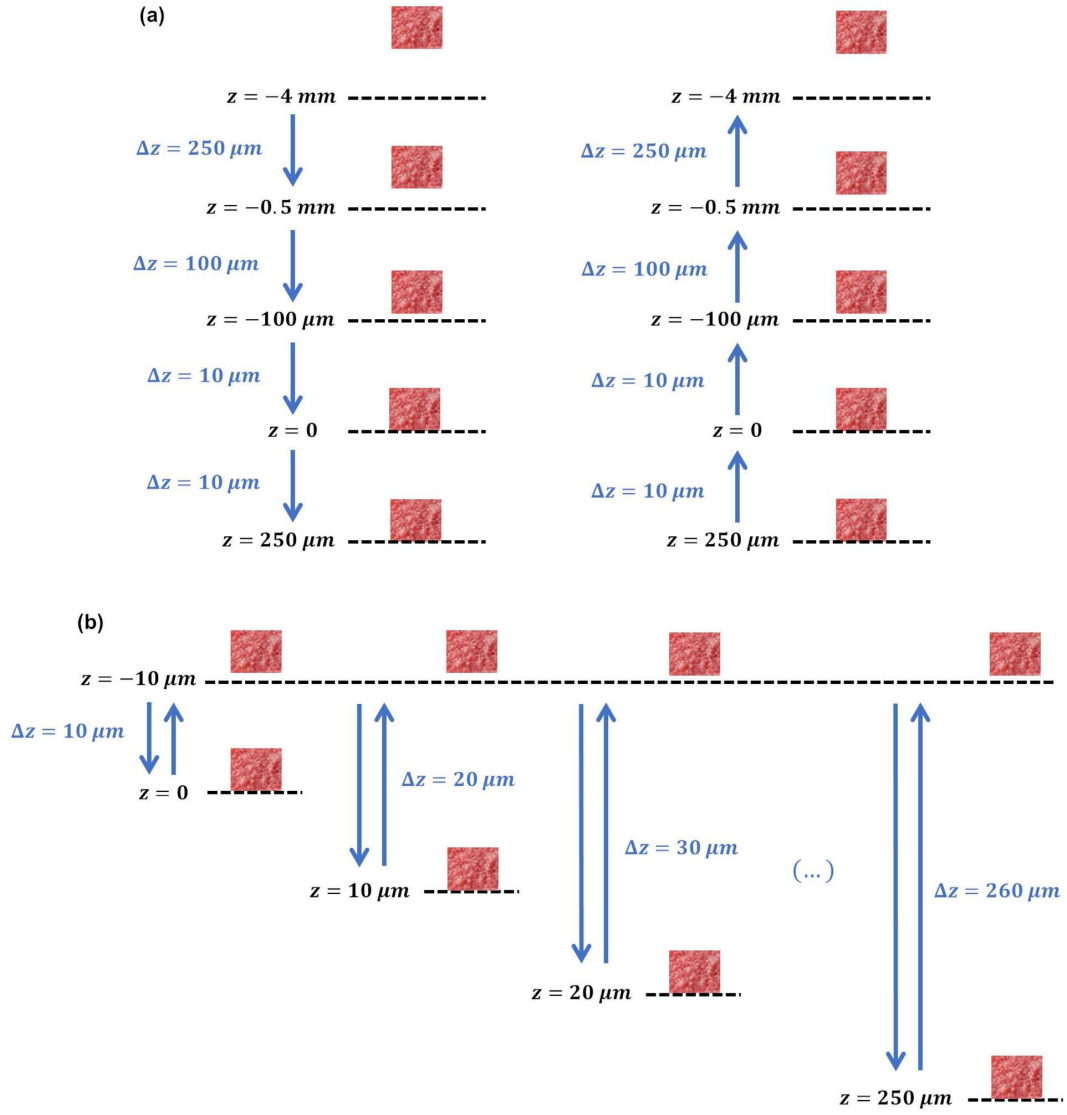
Experimental procedures for: (**a**) characterizing the capacitive change for different sensor-bone distances; (**b**) characterizing the bone-sensor contact patterns.

Two experimental procedures were performed: (1) characterization of the capacitive changes for different sensor-bone distances (Fig. 7a); (2) characterization of the contact patterns on the sensor-bone interface (Fig. 7b). Different sensor-bone distances were achieved by vertical step displacements ($\Delta z$) of the bone samples. The contact (interface in bonding states) and contactless (interface in loosening states) scenarios occur for $z\ge 0$ and $z<0$, respectively. Bone-sheet contacts were detected when desired displacements $\Delta z$ required compressive or decompressive loads over the bone samples.

Capacitive changes were measured by performing two different vertical trajectories. Firstly, a descending vertical trajectory was carried out in which $\Delta z$ was shortened as the bone samples approached the sensor-bone interface (Fig. 7a): starting at $z=-4$ mm (4 mm apart from the interfacial sheet, 5 mm apart from the capacitive sensor), $\Delta z = 250$ $\upmu \text {m}$ was defined up to $z=-500$ $\upmu \text {m}$; $\Delta z$ was shorten to 100 $\upmu \text {m}$ in the range $-500$ $\upmu \text {m}<z\le -100$ $\upmu \text {m}$; during the last stage without loading, $\Delta z$ was shorten to 10 $\upmu \text {m}$ until the bone-sheet contact was reached (1 mm apart from the capacitive sensor); $\Delta z=10$ $\upmu \text {m}$ was also used for contacts ranged between $0<z\le 250$ $\upmu \text {m}$, range in which compression tests were performed. When $z=250$ $\upmu \text {m}$ was reached, an ascending vertical trajectory was performed using the same $\Delta z$ as those used throughout the descending trajectory, although $\Delta z$ was enlarged as the bone samples were moved away from the sensor-bone interface (Fig. 7a): during decompressive loading, as well as in the range $0<z\le -100$ $\upmu \text {m}$ (the first stage without loading), $\Delta z = 10$ $\upmu \text {m}$ was defined; $\Delta z = 100$ $\upmu \text {m}$ and $\Delta z = 250$ $\upmu \text {m}$ were performed for $-500$ $\upmu \text {m}<z\le -100$ $\upmu \text {m}$ and $-4$ $\text {mm}<z\le -500$ $\upmu \text {m}$, respectively.

**Figure 8:**
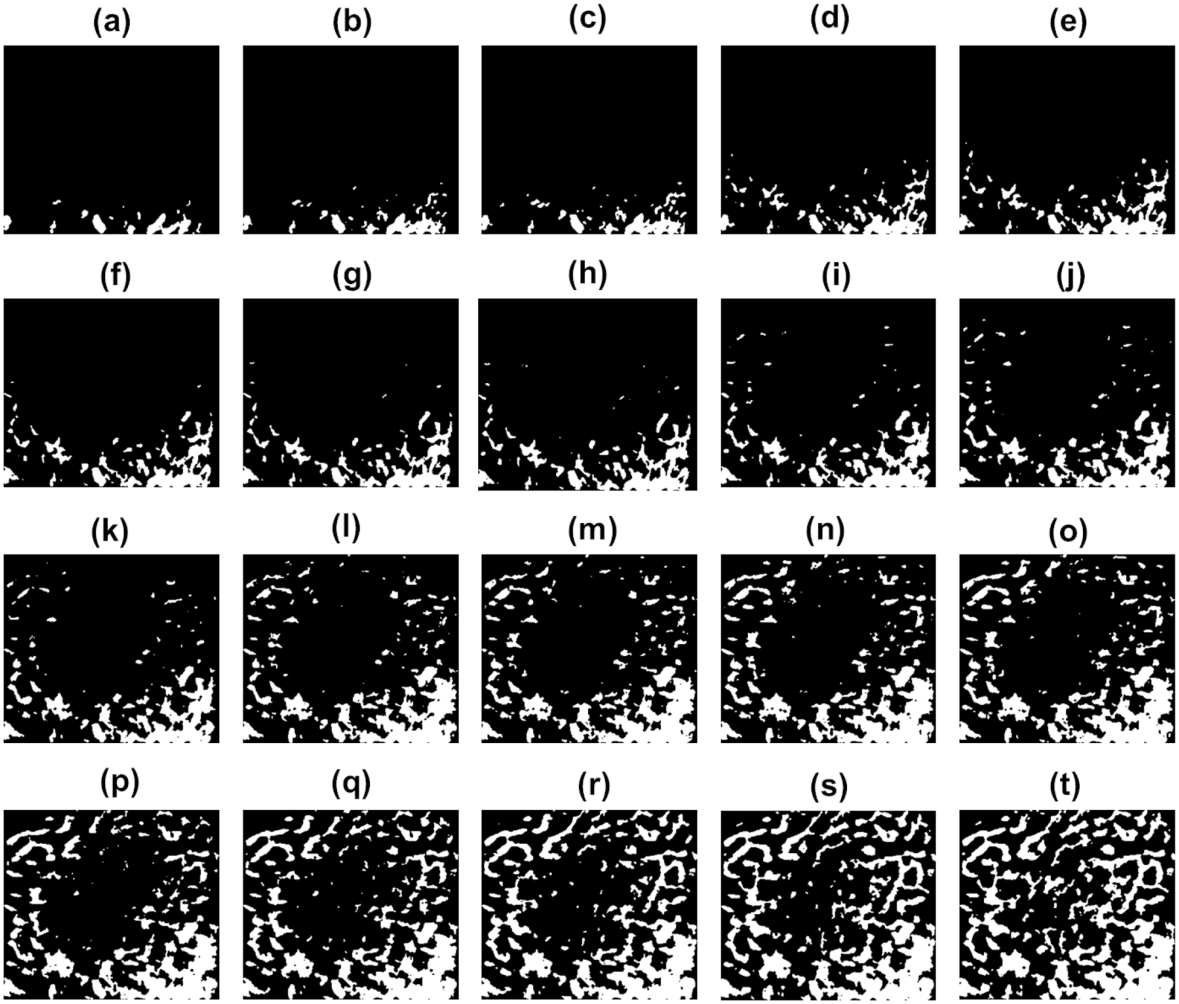
Characterization of bone-sheet contact patterns for the striped architecture (contact areas as a function of displacement *z*): (**a**) 2.34%, $z\approx 58$ $\upmu \text {m}$; (**b**) 3.87%, $z\approx 75$ $\upmu \text {m}$; (**c**) 4.24%, $z\approx 78$ $\upmu \text {m}$; (**d**) 4.77%, $z\approx 83$ $\upmu \text {m}$; (**e**) 5.24%, $z\approx 87$ $\upmu \text {m}$; (**f**) 5.94%, $z\approx 92$ $\upmu \text {m}$; (**g**) 6.22%, $z\approx 95$ $\upmu \text {m}$; (**h**) 6.89%, $z\approx 99$ $\upmu \text {m}$; (**i**) 8.10%, $z\approx 108$ $\upmu \text {m}$; (**j**) 12.1%, $z\approx 131$ $\upmu \text {m}$; (**k**) 16%, $z\approx 151$ $\upmu \text {m}$; (**l**) 19.3%, $z\approx 165$ $\upmu \text {m}$; (**m**) 21.6%, $z\approx 174$ $\upmu \text {m}$; (**n**) 23.3%, $z\approx 180$ $\upmu \text {m}$; (**o**) 27.5%, $z\approx 196$ $\upmu \text {m}$; (**p**) 29.4%, $z\approx 202$ $\upmu \text {m}$; (**q**) 30.6%, $z\approx 206$ $\upmu \text {m}$; (**r**) 32.3%, $z\approx 211$ $\upmu \text {m}$; (**s**) 34.6%, $z\approx 219$ $\upmu \text {m}$; (**t**) 36.2%, $z\approx 223$ $\upmu \text {m}$ (near the maximum bonding state). Similar contact patterns were found for other bone samples, including those used for the circular architecture.

Contact patterns of trabeculae on the interfacial sheet (samples under compressive loading) were observed by increasing $\Delta z$ (10 $\upmu \text {m}$ increasing steps) throughout a descending vertical trajectory in the range $-10$ $\upmu \text {m}\le z\le 250$ $\upmu \text {m}$, taken $z = 10$ $\upmu \text {m}$ as the starting reference (Fig. 7b). These contact patterns are shown in Fig. 8. Contact areas from a very weak bonding state of the organic phase (2.34%) to the maximum bonding state (36.2%) were monitored. A polynomial approximation ($R^2=0.9939$) of the contact areas $T_a$ as a function of displacement *z* can be computed by Eq. (1).

1 $$\begin{aligned} T_a(z)=\sum _{i=0}^{3} p_{tc_i} z^i, \qquad 0\le z\le 250\qquad [\upmu \text{m}] \end{aligned}$$

where $p_{tc_0} = -0.0326$, $p_{tc_1} = 48.73$, $p_{tc_2} = 6.804\times 10^{8}$ and $p_{tc_3} = 2.082\times 10^{11}$. This equation was empirically found using the experimental data observed from contact patterns shown in Fig. 8. It was used for developing the computational models described in the next section.

### Computational models

**Figure 9:**
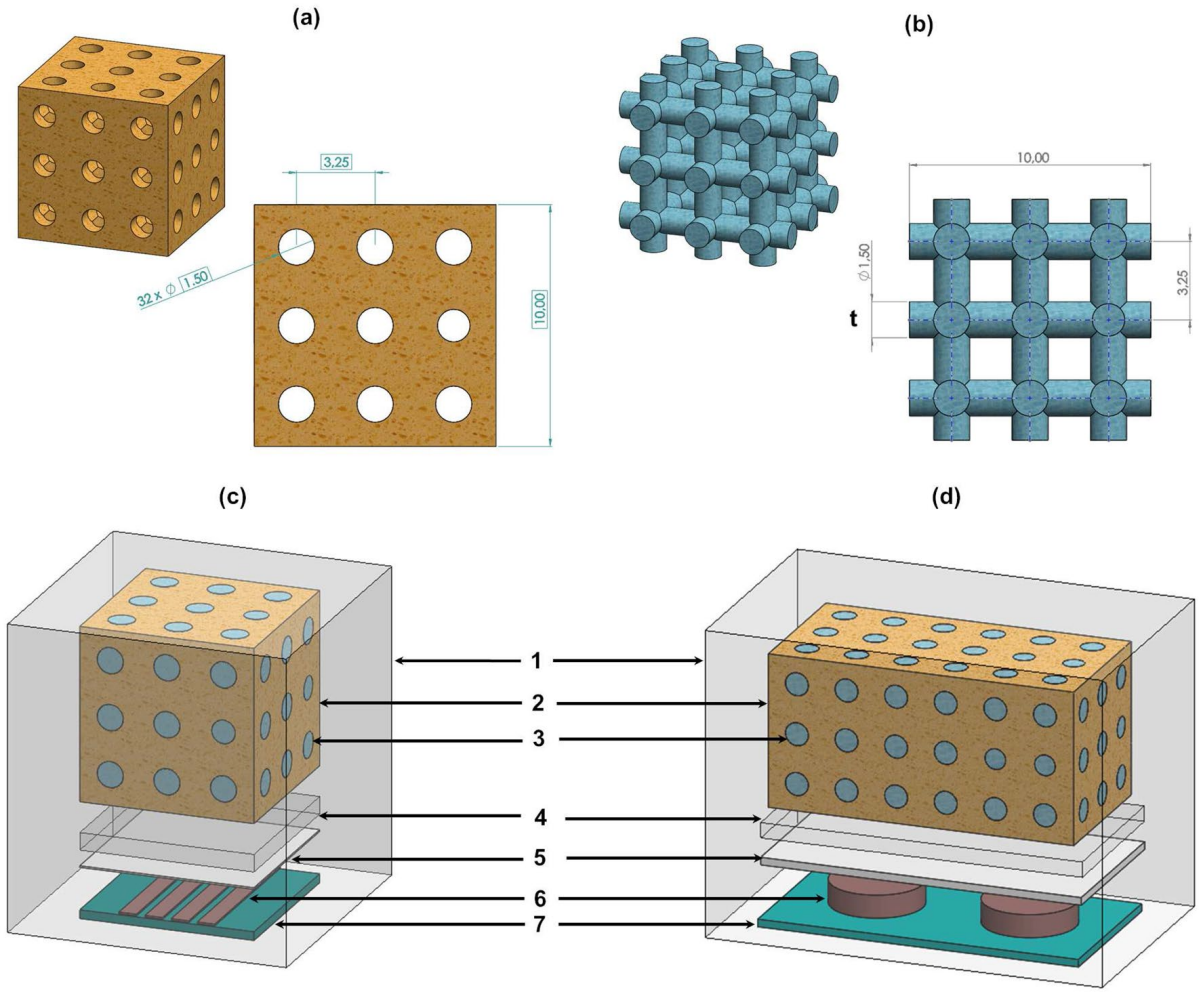
Domains of computational models to predict capacitive changes for bone-sensor interface changes: (**a**) liquid domain for the striped architecture (contactless scenarios); (**b**) trabeculae domain for the striped architecture (contactless scenarios). Similar domains were defined for the circular architecture, although with larger lengths (20 mm); (**c**,**d**) Model apparatuses for the striped and circular architectures, respectively, including the domain ’intermediate air’ (exploded view). Domains: 1—surrounding air; 2—liquid (bone); 3—trabeculae; 4—surrounding air; 5—interfacial sheet; 6— electrodes; 7—substrate.

Four finite element computational models were developed to numerically predict interface states between maximum bonding ($z=0.25$ mm) to maximum loosening ($z=-4$ mm): simplified models of striped and circular architectures for contactless scenarios ($-4\le z<0$ mm) and simplified models of striped and circular architectures for contact scenarios ($0\le z\le 250$ $\upmu \text {m}$). Bone samples were modelled as decalcified trabecular structures, according to a simplified biphasic porous structure, as illustrated in Fig. 9a,b, in which the trabeculae phase was designed as a structure to fulfil the interconnected pore network within liquid. Contactless scenarios were modelled with 35% trabeculae and 65% liquid; the contact scenarios were differently modelled: the trabecular content was adjusted to match the trabeculae contact areas experimentally observed on the interfacial sheet for different bonding states (Fig. 8). This latter simplified models were developed by redefining the parameter *t* (Fig. 9b) to match the trabecular contact area $T_a$ expressed by Eq. (1). Only trabecular contact areas higher than 16% were considered, corresponding to the lower limit from which trabecular contacts are uniformly distributed through the interfacial sheet (the trabecular contacts of the simplified bone models are also uniformly distributed). By using simplified models, not only computational simulation costs are significantly reduced, but also high correlations between simulation and experimental results may be achieved. If such high correlations are ensured, clinicians can follow-up the interface states without the need to consider all the realistic complexity of bone structures, namely the inhomogeneity of trabecular structures (resulting from crystalline and amorphous mineral and organic phases), as well as the quite complex strain effects occurring on trabeculae due to compressive and decompressive loading^5,42^.

All models were implemented according to a modelling methodology recently validated in silico and in vitro to analyse the performance of electric stimulation systems using cosurface capacitive architectures incorporated within bioelectronic implants^5,19,30^. Simplified models for contactless interfaces comprise six domains: surrounding air, substrate, electrodes, interfacial sheet, trabeculae and liquid. An additional domain, the intermediate air, was included in simplified models for contact interfaces. Substrate and interfacial sheet were defined as polymeric^5,19^, striped-shaped electrodes as copper and circular-shaped electrodes as Ni-Fe. Domains were organized as follows (Fig. 9c,d):

- Simplified models to simulate contactless interfaces: electrodes were positioned over the substrate and under the interfacial sheet, which in turn is under the bone sample;
- Simplified models to simulate contact interfaces: they are similar to those developed for contactless interfaces, but the intermediate air between the bone sample and the interfacial sheet was included.

Simulation details are provided in “Methods” section.

### Experimental performance

**Figure 10:**
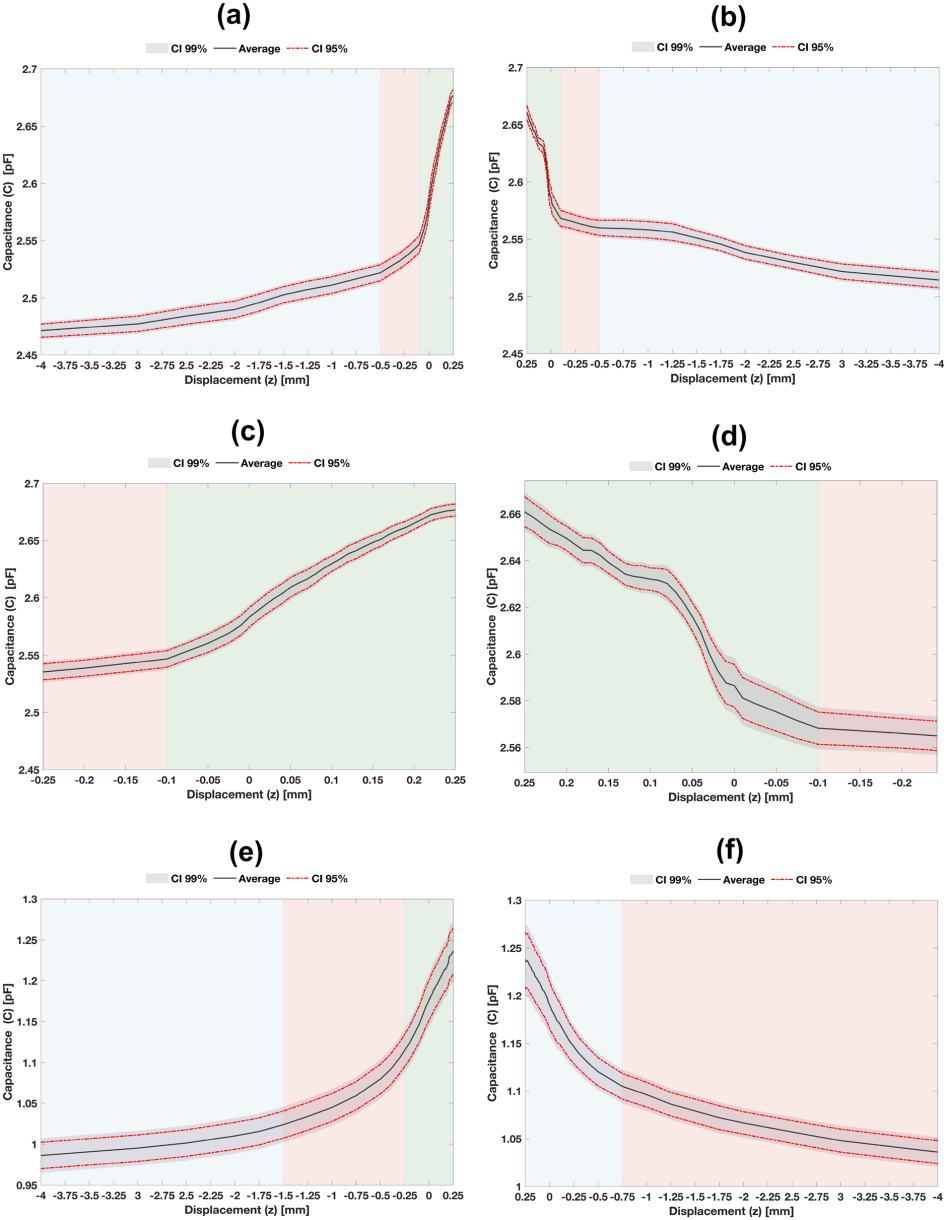
Experimental results in vitro: (**a**,**b**) Capacitance measured throughout the descending vertical trajectory ($-4 \rightarrow 0.25$ mm) and ascending vertical trajectory ($0.25 \rightarrow -4$ mm), respectively, using the striped cosurface architecture; (**c**,**d**) Detailed view of results shown in (**a**,**b**): descending trajectory in the range $-0.25\le z\le 0.25$ mm; ascending trajectory in the range $0 \le z\le 0.25$ mm; (**e**,**f**) Capacitance measured throughout the descending vertical trajectory ($-4 \rightarrow 0.25$ mm) and ascending vertical trajectory ($0.25 \rightarrow -4$ mm), respectively, using the circular cosurface architecture.

Similar trends were found for both the descending and ascending vertical trajectories, regardless the architectures, geometries and materials of cosurface capacitive sensors (Fig. 10). Capacitance varied according to similar increasing trends as the bone samples approach the striped or circular cosurface sensors (Fig. 10a,e). Differently, similar decreasing trends are observed for increasing bone-sensor distances (Fig. 10b,f). The most significant capacitive changes occur for interfaces close to the bone-sheet contact (pre-contact region) and just after the contact (post-contact region). Besides, minimum confidence intervals (CI) were found in regions between pre- and post-contacts. Therefore, these results highlight that these capacitive sensors are able to detect early states of loosening. Moreover, larger loosening can be also detected. The striped cosurface architecture demonstrated higher sensitivity both in pre- and post-contact regions, whereas the circular architecture provided higher capacitance variations from maximum bonding ($z=0.25$ mm) to maximum loosening ($z=-4$ mm), as shown in Fig. 10a,b,e,f).

Concerning the performance of the striped cosurface architecture during descending trajectories (Fig. 10a,c), the average capacitance variation was 0.2047 pF in the overall range $-4\le z\le 0.25$ mm, i.e., 48 $\text {aF}/\upmu \text {m}$. The following region-dependent average capacitance variations were measured: 50 fF for $-4\le z \le -0.5$ mm (24.43% increase; 14.3 $\text {aF}/\upmu \text {m}$), 25 fF for $-500<z\le -100$ $\upmu \text {m}$ (12.21% increase; 62.5 $\text {aF}/\upmu \text {m}$), 0.1297 pF for $-100<z\le 250$ $\upmu \text {m}$ (63.36% increase; 0.37 $\text {fF}/\upmu \text {m}$). Minimum deviations occurred when $z\approx 100$ $\upmu \text {m}$ (post-contact region; 99% CI $\pm 6.7$ fF; 95% CI $\pm 5.1$ fF), whereas maximum deviations were found when $z\approx -2.25$ mm (a loosening state; 99% CI $\pm 11.5$ fF; 95% CI $\pm 8.7$ fF). Capacitance saturated when the displacement *z* was increased beyond 250 $\upmu \text {m}$, i.e., when the maximum bonding was achieved ($\approx 36\%$ contact area; Fig. 8t), even when bone samples were loaded with higher compressive forces. Saturation occurred at $\approx 14$ N. This phenomenon was observed for both architectures. The average capacitance ($C_{sd}$) exhibited a polynomial behavior expressed by Eq. (2) ($R^2 = 0.988$).

2 $$\begin{aligned} C_{sd}(z)=\sum _{i=0}^{6} p_{sd_i} z^i, \qquad -4\le z\le 0.25\quad [\text{mm}] \end{aligned}$$

where $$p_{sd_0} = 2.588\times 10^{-12}$$, $$p_{sd_1} = 2.925\times 10^{-10}$$, $$p_{sd_2} = 4.217\times 10^{-7}$$, $$p_{sd_3} = 2.894\times 10^{-4}$$, $$p_{sd_4} = 9.628\times 10^{-2}$$, $$p_{sd_5} = 14.95$$ and $$p_{sd_6} = 850.2$$.

The striped cosurface architecture also provided a well-defined performance throughout ascending trajectories (Fig. 10b,d). 0.1465 pF of average capacitance variation was measured for bone-sheet distances ranged between $-4\le z\le 0.25$ mm (34.5 $\text {aF}/\upmu \text {m}$). The average capacitance variations were also found region-dependent: 18.6 fF for $-4\le z \le -0.5$ mm (12.7% increase; 5.3 $\text {aF}/\upmu \text {m}$), 9.1 fF for $-500<z\le -100$ $\upmu \text {m}$ (6.21% increase; 22.7 $\text {aF}/\upmu \text {m}$), 0.1188 pF for $-100<z\le 250$ $\upmu \text {m}$ (81.09% increase; 0.34 $\text {fF}/\upmu \text {m}$). Therefore, although a lower capacitance variation between maximum bonding to maximum loosening ($-4\le z \le -0.5$ mm) is provided by ascending trajectories, higher variations in the pre- and post-contact regions are observed. Minimum and maximum deviations were measured for $z=0$ (minimum bonding state; 99% CI $\pm 12$ fF; 95% CI $\pm 9.1$ fF) and $z\approx -1$ mm (a loosening state; 99% CI $\pm 24.1$ fF; 95% CI $\pm 18.3$ fF), respectively. The average capacitance behavior ($C_{sa}$) was also analytically modelled by a polynomial function, as follows (Eq. 3; $R^2 = 0.965$).

3 $$\begin{aligned} C_{sa}(z)=\sum _{i=0}^{6} p_{sa_i} z^i, \qquad -4\le z\le 0.25\quad [\text{mm}] \end{aligned}$$

where $$p_{sa_0} = 2.597\times 10^{-12}$$, $$p_{sa_1} = 1.989\times 10^{-10}$$, $$p_{sa_2} = 3.294\times 10^{-7}$$, $$p_{sa_3} = 2.387\times 10^{-4}$$, $$p_{sa_4} = 7.988\times 10^{-2}$$, $$p_{sa_5} = 12.1$$ and $$p_{sa_6} = 649$$.

Analysing the sensing performance of the circular cosurface architecture (Fig. 10e), 0.2498 pF of average capacitance variation was found during descending experiments for $-4\le z\le 0.25$ mm (58.8 $\text {aF}/\upmu \text {m}$). Three main regions were identified with different average capacitance variation rates: 37.8 fF for $-4\le z \le -1.5$ mm (15.13% increase; 15.1 $\text {aF}/\upmu \text {m}$), 91 fF for $-1500<z\le -250$ $\upmu \text {m}$ (36.43% increase; 72.8 $\text {aF}/\upmu \text {m}$), 0.121 pF for $-250<z\le 250$ $\upmu \text {m}$ (48.44% increase; 0.242 $\text {fF}/\upmu \text {m}$). When the bone samples were positioned at $z\approx -50$ $\upmu \text {m}$ (pre-contact region), minimum deviations were found (99% CI $\pm 15.4$ fF; 95% CI $\pm 16.1$ fF). Maximum deviations were observed at $z=-4$ mm (maximum loosening state; 99% CI $\pm 37.6$ fF; 95% CI $\pm 28.6$ fF). Equation (4) was used to compute the average capacitance ($C_{cd}$) as a function of the displacement *z* ($R^2 = 0.998$).

4 $$\begin{aligned} C_{cd}(z)=\sum _{i=0}^{4} p_{cd_i} z^i, \qquad -4\le z\le 0.25\quad [\text{mm}] \end{aligned}$$

where $$p_{cd_0} = 1.172\times 10^{-12}$$, $$p_{cd_1} = 235.6\times 10^{-10}$$, $$p_{cd_2} = 1.386\times 10^{-7}$$, $$p_{cd_3} = 3.805\times 10^{-5}$$ and $$p_{cd_4} = 3.807\times 10^{-3}$$.

Finally, 0.202 pF of average capacitance variation (47.5 $\text {aF}/\upmu \text {m}$) was found using the circular cosurface architecture during ascending experiments between maximum bonding to maximum loosening ($-4\le z \le -0.5$ mm), as shown in Fig. 10f, which is also a lower capacitance variation when compared to descending trajectories. Based on a region-dependent analysis, two main regions were identified with differing average capacitance variations: 69.1 fF for $-4\le z \le -0.75$ mm (34.21% increase; 21.3 $\text {aF}/\upmu \text {m}$), 132.9 fF for $-750<z\le 250$ $\upmu \text {m}$ (65.79% increase; 132.9 $\text {aF}/\upmu \text {m}$). Minimum deviations were found at the pre-contact region ($z\approx -100$ $\upmu \text {m}$; 99% CI $\pm 15.4$ fF; 95% CI $\pm 11.7$ fF); differently, maximum deviations were detected when the maximum bonding states occur ($z=250$ $\upmu \text {m}$; 99% CI $\pm 37.6$ fF; 95% CI $\pm 28.6$ fF). The average capacitance ($C_{ca}$) was also analytically modelled for different bone-sheet distances, as described by Eq. (5) ($R^2 = 0.998$).

5 $$\begin{aligned} C_{ca}(z)=\sum _{i=0}^{4} p_{ca_i} z^i, \qquad -4\le z\le 0.25\quad [\text{mm}] \end{aligned}$$

where $p_{ca_0} = 1.019\times 10^{-12}$, $p_{ca_1} = 1.832\times 10^{-10}$, $p_{ca_2} = 1.154\times 10^{-7}$, $p_{ca_3} = 3.436\times 10^{-5}$ and $p_{ca_4} = 3.636\times 10^{-3}$. Equations (2) to (5) were found to provide simple analytical models that can be used instead of the complex numerical models.

### Simulation results

Simulation results were analysed by normalizing data in the range between 0 and 1, corresponding to the maximum loosening ($z=-4$ mm) and maximum bonding ($z=250$ $\upmu \text {m}$), respectively. Analyses were conducted after data normalization because: (i) simulation results do not include all capacitances influencing experimental results, such as capacitances due to conductive cables, connectors, welds and PCB tracks, among others; (ii) analyses using relative data are far more relevant than using absolute data, as absolute capacitances are deeply influenced by bone quality, bone density, anatomical region, animal model, etc.; (iii) experimental data can be easily (re)calibrated by additional electronic processing systems, such that the clinical follow-up of bone-sensor displacements can be ensured by analysing the capacitance variations from a relative value (e.g. from a bone-implant bonding state).

**Figure 11:**
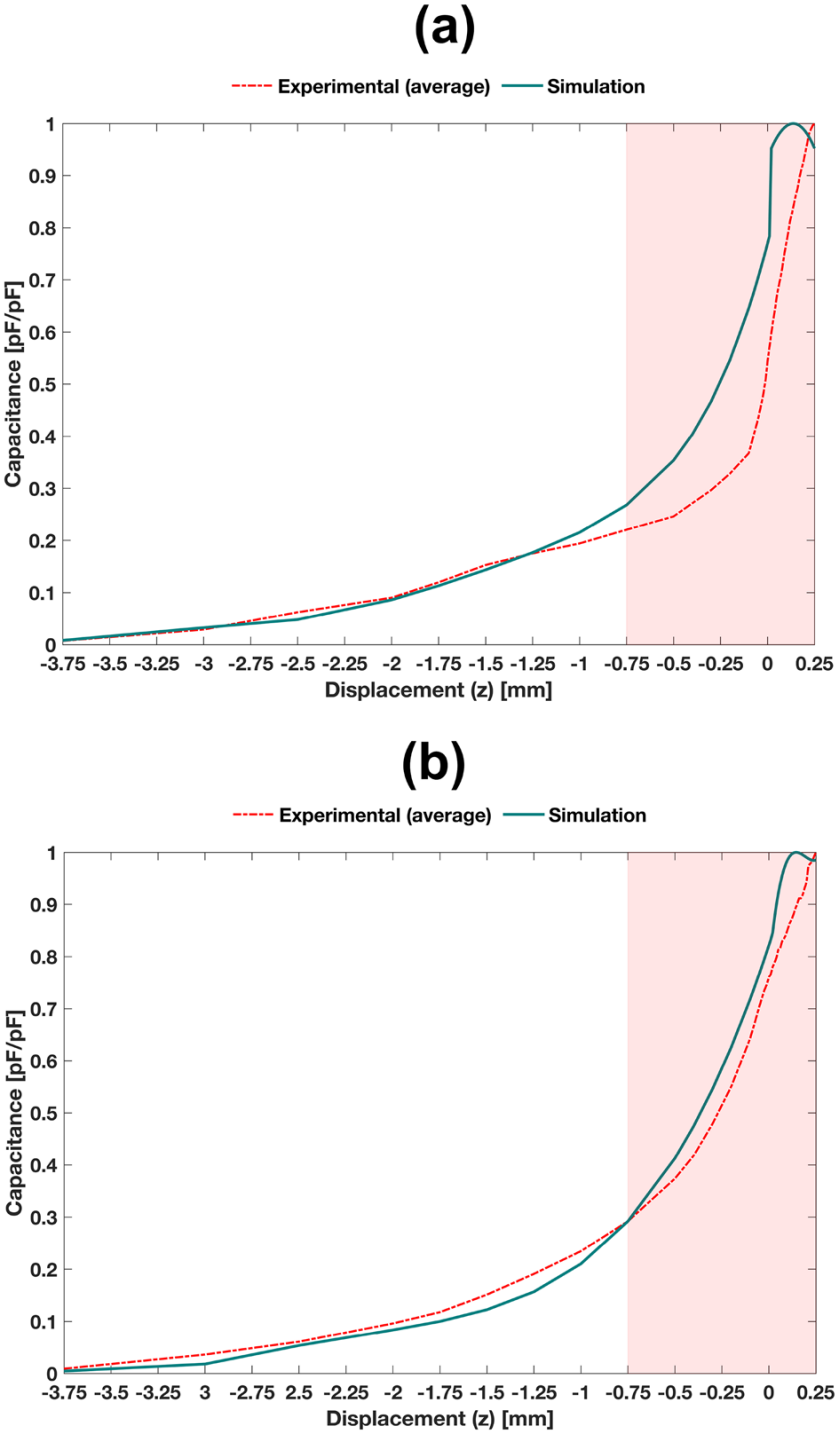
Simulation and experimental results: (**a**) striped architecture; (**b**) circular architecture. The experimental results here illustrated are the average capacitances computed using Eqs. (2) and (4).

Good agreement was obtained between numerical simulation and experimental results: correlations higher than 86% was achieved for $-4\le z\le 0.25$ mm for both the striped and circular architectures (Fig. 11). The circular architecture provided higher correlations than those provided by the striped architecture. These simulation results highlight that simplified models of decalcified bone structures are effective for predicting interface states.

Concerning the striped architecture (Fig. 11a), the following correlation results were obtained: overall correlation of 93.9%, p-value of 0, lower bound of 86.7% and upper bound of 97.2% for a 99% CI; for a 95% CI, the overall correlation was also 93.9%, the p-value was 0, the lower bound was 88.9% and the upper bound was 96.6%. These results emphasize that bone-implant interface states can be predicted from maximum bonding states ($z=250$ $\upmu \text {m}$; 36% trabeculae contact) to maximum loosening ($z=-4$ mm; no trabeculae contact). Analysing the correlation data in the range $-4\le z\le 0.01$ mm, even better agreements were found: correlation of 98.3%, p-value of 0, lower bound of 94.3% and upper bound of 99.5% for a 99% CI; for a 95% CI, the correlation was 98.3%, the p-value was 0, the lower bound was 95.7% and the upper bound was 99.4%. Although less accurate results were observed in the range $-750\le z \le 250$ $\upmu \text {m}$ (correlation for 99% CI: 97%; p-value: 0; lower bound: 67%; upper bound: 99.8%), simulation results exhibit an approximate general increasing trend to experimental results in vitro. The maximum error between simulation and experimental results was 35.4%, corresponding to an absolute maximum error of 1.48 pF. However, the capacitance variation patterns are significantly more relevant than absolute data, as absolute errors may be eliminated by electronic recalibration of capacitive sensors.

More accurate predictions were provided by computational models of circular architectures (Fig. 11b). The correlation in the overall range $-4\le z\le 0.25$ mm was 99.24%, p-value of 0, lower bound of 98.32% and upper bound of 99.66% for a 99% CI. Therefore, these models are able to provide valuable data concerning the bone-implant interface states in an extent ranging from maximum loosening states to maximum bonding states. Very high correlations were also achieved for $-4\le z\le 0.01$ mm: correlation of 99.82%, p-value of 0, lower bound of 99.36% and upper bound of 99.95% for a 99% CI. The region defined in the interval $-750\le z \le 250$ $\upmu \text {m}$ also presented a good agreement for 99% CI: correlation of 97.3%, p-value of 0; lower bound of 93.5% and upper bound of 98.89%. These results show that the increasing trend provided by the simulation results approaches the one provided by experimental results. A maximum error of 13.6% between simulation and experimental results was achieved, which highlights the very good accuracy of the computational models of circular architectures. 0.85 pF of absolute maximum error was obtained, which is about half the error obtained by the striped architecture.

## Discussion

**Figure 12:**
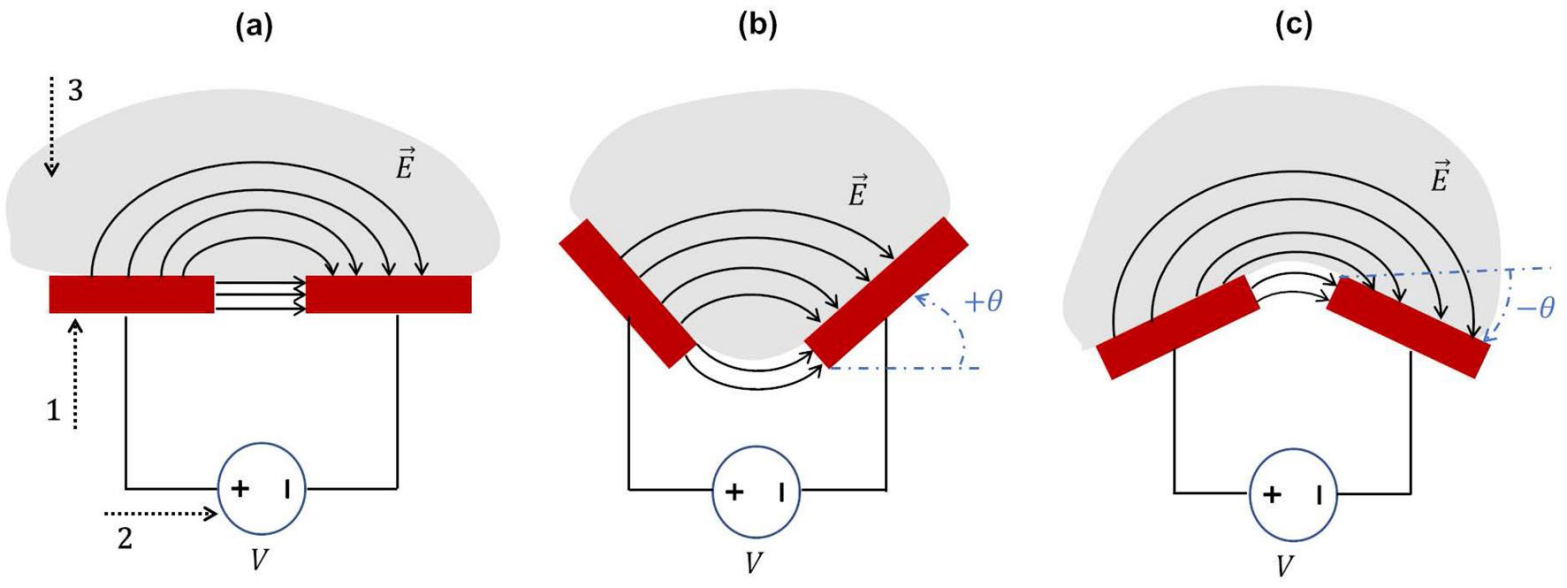
Influence of capacitive sensing system inclination on electric field stimuli: (**a**) without inclination (according to the setup analysed in this study); (**b**) angle inclination $+\theta$ (embracing the bone structures); (**c**) angle inclination $-\theta$. Legend: 1—electrodes; 2—eletric power source; 3—bone structures.

A multifaceted study here presented includes: (i) the introduction of a novel concept of cosurface capacitive sensing to monitor bone-implant interface states inside bioelectronic implants; (ii) the first experimental tests in vitro using different bone-sensor interfaces; and (iii) the use of numerical models to predict both loosening or bonding states. The sensing performance of two capacitive technologies were analysed: the striped and circular architectures. Experimental and simulation results demonstrate that both architectures are effective detecting the interface state from maximum bonding states to significant loosening states, even though they were designed with different geometries and materials. Unlike most of technologies developed so far^35^, the ability of these capacitive sensors is neither limited to detect only few loosening states nor unable to detect the loosening magnitude. The cosurface capacitive technologies present a highly improved performance over all other technologies already proposed because they can detect micro-scale and macro-scale bonding, debonding or loosening, mainly when the bone-implant bonding is weakening or when early states of loosening occur. Indeed, they fulfill the six main requirements for effective monitoring^35^: (1) operate noninvasively; (2) allow incorporation inside bioelectronic implants; (3) allow miniaturized, stretchable and flexible integration inside implants; (4) allow customized designing for different topological structures and geometries; (5) provide controllable and personalized monitoring of target peri-implant tissues; (6) allow the follow-up of the bone–implant fixation states throughout the daily life of patients (Table 1). The striped capacitive sensor presented higher sensitivity detecting this small-scale debonding disorders, although the circular capacitive system provided higher capacitance variations detecting large-scale loosenings. It is noteworthy that different conclusions could be found if the striped technology were manufactured with Ni-Fe electrodes and the circular technology with copper electrodes, although they most likely would exhibit similar sensing performances. Higher capacitive changes will be achieved if larger electrodes and narrowed gaps (between electrodes) are used. On the one hand, the use of larger electrodes does not allow the miniaturization of the sensing systems; on the other hand, gap minimization contributes for engineering a miniaturized technological solution. So, a compromise solution must be found. Different behaviours from the ones here presented may occur for different inclinations of the capacitive architectures (Fig. 12): (a) for bioelectronic implants engineered with electrodes positioned with positive inclinations (electrodes embracing the bone structures; Fig. 12b), higher capacitive changes are expected for the same bone loss scenario; (b) for bioelectronic implants engineered with electrodes positioned with negative inclinations (electrodes not embracing the bone structures; Fig. 12c), lower capacitive changes will most likely occur for the same bone loss scenario. In this later apparatus, high sensing performance will most likely be obtained if a network of miniaturized sensing systems is used to ensure low inclinations of the electrodes along the implant surface: a *quasi*-horizontal positioning of the electrodes along the implant surface will be achieved if they are miniaturized. Interface states can be monitored using experimental data and, surprisingly, using simulation data from simplified models of decalcified bone structures. Computational models developed in this study are more suitable for therapeutic actuation (aiming to eliminate or minimize a loosening state) than for prophylactic atuation. Negligible differences in simulation results were found using computational models developed in such a way that bone samples were laid with very tiny inclinations according to contact patterns experimentally observed (Fig. 8).

**Table 1:** Comparative analysis of the monitoring methods already proposed to fulfil the effectiveness criteria^a^.

Methodologies	Fixation	Methods	Requirements					
			(1)	(2)	(3)	(4)	(5)	(6)
Vibrometric	Cementless	Ext. mechanical excitation–ext. mechanical output	$\checkmark$			$\checkmark$		$\checkmark$
		Ext. magnetic induction–ext. mechanical output	$\checkmark$	$\checkmark$		$\checkmark$		$\checkmark$
		Int. mechanical excitation–ext. mechanical output	$\checkmark$			$\checkmark$		$\checkmark$
	Cemented	Ext. mechanical excitation–ext. mechanical output	$\checkmark$			$\checkmark$		$\checkmark$
		Ext. mechanical excitation–int. mechanical output	$\checkmark$	$\checkmark$		$\checkmark$		$\checkmark$
Acoustic	Cementless	Ext. mechanical excitation–ext. acoustic signal	$\checkmark$			$\checkmark$		$\checkmark$
		Int. mechanical excitation–ext. acoustic output	$\checkmark$			$\checkmark$		$\checkmark$
		Ext. magnetic induction–ext. acoustic output	$\checkmark$	$\checkmark$		$\checkmark$		$\checkmark$
	Cemented	Ext. mechanical excitation–ext. acoustic output	$\checkmark$			$\checkmark$		$\checkmark$
		Int. mechanical excitation–ext. acoustic output	$\checkmark$			$\checkmark$		$\checkmark$
		Int. mechanical excitation–int. acoustic output	$\checkmark$	$\checkmark$		$\checkmark$		$\checkmark$
		Ext. acoustic excitation–ext. acoustic output	$\checkmark$			$\checkmark$		
Bioelectric impedance	Cementless	Ext. current excitation–ext. voltage output	$\checkmark$			$\checkmark$		$\checkmark$
Magnetic induction	Cementless	Ext. magnetic induction excitation–ext. magnetic induction output	$\checkmark$	$\checkmark$		$\checkmark$		$\checkmark$
Strain	Cementless	Int. mechanical loading excitation–int. deformation output		$\checkmark$	$\checkmark$	$\checkmark$		$\checkmark$
		Int. mechanical loading output–int. deformation output	$\checkmark$	$\checkmark$	$\checkmark$	$\checkmark$		$\checkmark$
Cosurface capacitance	Cementless	Int. voltage excitation–int. capacitance output	$\checkmark$	$\checkmark$	$\checkmark$	$\checkmark$	$\checkmark$	$\checkmark$

^a^Terminology: Int.—intracorporeal; Ext.—extracorporeal.

As these capacitive sensors require very low electric currents and voltage amplitudes not exceeding 5 V, extracorporeal powering^35,40,41^ can be replaced by energy harvesting inside bioelectronic implants^6,43^. Another significant advantage is the ability of the same cosurface technologies to provide both sensing and therapeutic actuation operations^5,19^. Processing systems incorporated inside instrumented implants can be designed to control both the personalized delivery of electric stimuli to peri-implant target tissues, as well as the personalized monitoring of the same tissues. The ultimate goal is to engineer a technology such that clinicians can communicate with the implant after surgery: they will be permissions to control the implant, namely to define the electric stimuli to deliver to each region of the bone-implant interface, according to monitored data acquired by capacitive sensors positioned along the implant surface^23^. Therefore, the performance of multifunctional bioelectronic implants will be strongly improved if they are designed incorporating these capacitive architectures.

Future research must be conducted to analyse the impact of more realistic scenarios of peri-prosthetic bone boss on the performance of this capacitive sensing technology, namely the influence of fibrous tissues, liquid phases and hydroxyapatite-collagen structures. Temperature profiles in the capacitance variation must also be researched, namely for temperatures in the range between 37 and 43 °C, as already measured inside instrumented implants during in vivo tests^44,45^. Computational models for ascending trajectories must be developed to simulate the sensor performance during prophylactic atuation. Besides, models of striped achitectures for descending trajectories must be optimized to provide very good accurate simulation results. The design and manufacture of bioelectronic implants embedding cosurface capacitive sensors is also envisaged for in vitro and in vivo tests. Indeed, although results here reported are promising, clinical validation is required to demonstrate the effectiveness of the cosurface capacitive technologies to monitor the bone-implant interface. Clinical trials may reveal a specific behaviour for each specific group of patients (such as age-related patients, comorbidity-related patients, etc.), but the general behaviour will most likely be similar to the one found in this study (Fig. 11). Although intracorporeal power generation will allow to electrically supply these sensors throughout the daily life of patients^35^, the capacitance-to-digital converter must be miniaturized and its power requirements minimized. Finally, miniaturized processing systems must be implemented to allow both personalized monitoring and stimulation of the peri-implant tissues.

## Methods

### Manufacture of capacitive sensors

Electrodes from the striped architecture were fabricated in copper using PCB technology. The Ni-Fe alloy used for the circular architecture was the permalloy ASTM A753 Alloy Type 4 ($\text {M}\mu \text {Shield}$, Manchester, NH, USA)^46^. Wire connections from this architecture were also implemented using PCB technology.

### Monitoring apparatus

**Figure 13:**
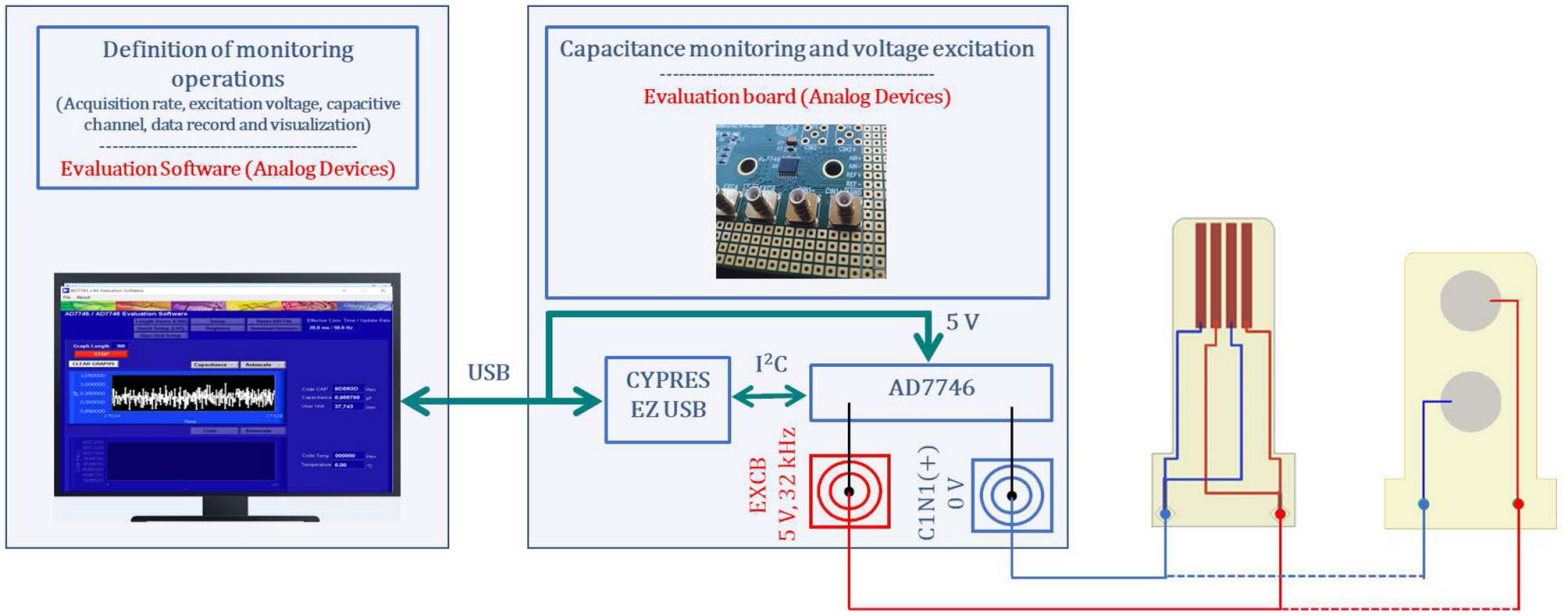
Schematics of the overall sensing system to monitor capacitance changes in bone-sensor interfaces (red wires and connectors: positively charging the electrodes; blue wires and connectors: negatively charging the electrodes).

A 24-bit capacitance-to-digital converter AD7746 (Analog Devices, Norwood, MA, EUA) was used to measure capacitance changes for different bone-sensor interfaces. This hardware was chosen as it provides 4 aF ($4\times 10^{-18}\text {F}$) of resolution, 4 fF ($4\times 10^{-15}\text {F}$) of accuracy, 0.01% of linearity, $\pm 4$ pF ($4\times 10^{-12}\text {F}$) of full-scale changing capacitance range and update rate up to 90 Hz. It is integrated into the platform EVAL-AD7746EB (Analog Devices), which includes an evaluation board for the AD7746 and the Evaluation Software (v2.2, Analog Devices), which runs on a PC to control the monitoring operations and perform data acquisition. Communication between the PC and the converter AD7746 was established by a USB interface and $\text {I}^2\text {C}$ protocol. Capacitive architectures were electrically connected to the AD7746 converter using a RG174 coaxial cable and connectors C1N1 (positive charge) and EXCB (negative charge) of the evaluation board, as illustrated in Fig. 13. The converter was configured in the single-ended mode. Capacitance measures were performed at 50 Hz (20 ms acquisition time). Three hundred capacitance measures were acquired for each interface state to allow steady-state capacitance analyses (transient responses were rejected).

### Experimental tests

The universal testing machine Trapezium X (Shimadzu) was used. An aluminium board (structure D; Fig. 4b) was included to fix the structure C to the testing machine, as well as to provide mechanical stability during experimental tests. The high resolution camera U3-3480ML (IDS, Obersulm, Germany) was used, as well as the UC Series lens with 4mm fixed focal length (Edmund Optics, NJ, USA). This camera provides a 1.31 Mpix resolution ($1280\times 1024$ pixel), 4.8 $\upmu \text {m}$ of pixel size and optical size of 6.144 $\text {mm}\times 4.915$ mm. The metallic powder was provided by the revealing spray Rotrivel U R2.82T (CGM CIGIEMME, Milan, Italy). The software IDS Vision Cockpit (v. 4.91, IDS) was used to acquire all contact patterns for different bone-sensor interfaces. Image processing was performed using the image processing toolbox of Matlab (v. 9.7, R2019b, Mathworks). All experimental in vitro tests were conducted at 22 °C and 50% humidity.

### Bone samples

Trabecular samples (Department of Veterinary Medicine, University of Évora, Portugal) of post-mortem porcine models were machined using a vertical milling machining LC-185 (First, Taiwan, China). Machined samples were firstly frozen; before experimental tests, they were defrosted for 15 min at 22 °C. For decalcification, the cubic-shaped and rectangular cuboid-shaped samples were fixed in 10% neutral-buffered formalin for 15 days, as previously established^47,48^.

### Simulation details

Table 2 presents the dimensions of each domain for both the striped and circular architectures, whereas the electric and magnetic properties of related materials are described in Table 3. Capacitance for different bone-sensor interfaces was computed using a multi-physics approach provided by COMSOL Multiphysics (v. 5.3, COMSOL). Domains were modelled as homogeneous and isotropic. They were tessellated by 3D meshes of tetrahedral linear elements of second order (Delaunay method). The domain ‘surrounding air’ was modelled using a fine mesh; all other domains were modelled using extra fine meshes. Mesh refinement and dimensioning of the ‘surrounding air’ were conducted by convergence analysis (2% error as stop criterion). 834553 and 768094 elements were required to tessellate the striped architecture for $z = -4$ mm and $z = 0.25$ mm, respectively; the circular architecture required 4955437 and 3923691 elements for $z = -4$ mm and $z = 0.25$ mm, respectively. The homogeneous Neumann condition was imposed to interior boundaries. External boundaries were magnetically isolated. Capacitance changes were simulated using the AC/DC module (physics interface: ‘Magnetic and Electric Fields’), which solved the Maxwell’s equations in the frequency-domain.

**Table 2:** Dimensions of domains used in computational models.

Domain	Striped architecture	Circular architecture
Bone sample	$10\times 10\times 10$ mm	$20\times 10\times 10$ mm
Intermediate air	$10\times 10\times z$ mm^a^	$20\times 10\times z^{(a)}$ mm
Interfacial sheet	$10\times 10\times 1$ mm	$20\times 10\times 1$ mm
Copper electrodes	$10\times 10\times 0.1$ mm	–
Ni-Fe electrodes	–	$\varnothing 6$ mm$\times 1.5$ mm
Substrate	$10\times 10\times 0.5$ mm	$20\times 10\times 0.5$ mm
Surrounding air	$16\times 16\times 28.5$ mm	$26\times 16\times 24$ mm

^a^$$0<z\le 4$$ mm.

**Table 3:** Electric and magnetic properties of organic and inorganic materials used in computational models for 32 kHz excitation^19,49,50^.

Domain	Relative electric permittivity	Electric conductivity $[\text {S/m}]$	Relative magnetic permeability
Trabeculae	$7.6\times 10^2-4.7\times 10^4j$	0.07	1
Liquid (bone)	$5.2\times 10^3-3.9\times 10^5j$	0.7	1
Intermediate air	1	0	1
Interfacial sheet	3	6.7 $\times$ $10^{-14}$	0.866
Copper electrodes	1	6 $\times$ $10^7$	1
Ni-Fe electrodes	1	1.64 $\times$ $10^6$	5 $\times$ $10^4$
Substrate	3	6.7 $\times$ $10^{-14}$	0.866
Surrounding air	1	0	1

Electrodes were powered by a pulsed waveform (50% duty cycle) with 5 V amplitude and 32 kHz frequency, defined as $V =\frac{V_{pp}}{2}+\frac{V_{pp}}{2}sign(\sin(2\pi f t))$, in which $V_{pp}$ refers to the pick-to-pick voltage and $f=32$ kHz. This excitation is the same as the one used for experimental tests. Capacitance was then obtained using Eq. (6).

6 $$\begin{aligned} C=\frac{-j \textit{Im}(Y)}{2\pi f} \end{aligned}$$

where $$f=32$$ kHz.

## Data Availability

The main data supporting the results in this study are available within the paper. The raw datasets here reported will be available upon request.

## Code availability

The custom computer code developed in this work is too big to be publicly shared, but will be available upon request.
